# KSAC, a Defined *Leishmania* Antigen, plus Adjuvant Protects against the Virulence of *L. major* Transmitted by Its Natural Vector *Phlebotomus duboscqi*

**DOI:** 10.1371/journal.pntd.0001610

**Published:** 2012-04-03

**Authors:** Regis Gomes, Clarissa Teixeira, Fabiano Oliveira, Phillip G. Lawyer, Dia-Eldin Elnaiem, Claudio Meneses, Yasuyuki Goto, Ajay Bhatia, Randall F. Howard, Steven G. Reed, Jesus G. Valenzuela, Shaden Kamhawi

**Affiliations:** 1 Vector Molecular Biology Section, Laboratory of Malaria and Vector Research, National Institutes of Allergy and Infectious Diseases, National Institutes of Health, Rockville, Maryland, United States of America; 2 Laboratory of Parasitic Disease, National Institutes of Allergy and Infectious Diseases, National Institutes of Health, Rockville, Maryland, United States of America; 3 Department of Zoology, Eastern Shore University, Eastern Shore Maryland, Maryland, United States of America; 4 Infectious Disease Research Institute, Seattle, Washington, United States of America; Louisiana State University, United States of America

## Abstract

**Background:**

Recombinant KSAC and L110f are promising *Leishmania* vaccine candidates. Both antigens formulated in stable emulsions (SE) with the natural TLR4 agonist MPL® and L110f with the synthetic TLR4 agonist GLA in SE protected BALB/c mice against *L. major* infection following needle challenge. Considering the virulence of vector-transmitted *Leishmania* infections, we vaccinated BALB/c mice with either KSAC+GLA-SE or L110f+GLA-SE to assess protection against *L. major* transmitted via its vector *Phlebotomus duboscqi*.

**Methods:**

Mice receiving the KSAC or L110f vaccines were challenged by needle or *L. major*-infected sand flies. Weekly disease progression and terminal parasite loads were determined. Immunological responses to KSAC, L110f, or soluble *Leishmania* antigen (SLA) were assessed throughout vaccination, three and twelve weeks after immunization, and one week post-challenge.

**Results:**

Following sand fly challenge, KSAC-vaccinated mice were protected while L110f-vaccinated animals showed partial protection. Protection correlated with the ability of SLA to induce IFN-γ-producing CD4^+^CD62L^low^CCR7^low^ effector memory T cells pre- and post-sand fly challenge.

**Conclusions:**

This study demonstrates the protective efficacy of KSAC+GLA-SE against sand fly challenge; the importance of vector-transmitted challenge in evaluating vaccine candidates against *Leishmania* infection; and the necessity of a rapid potent Th1 response against *Leishmania* to attain true protection.

## Author Summary

Leishmaniasis is a neglected disease caused by the *Leishmania* parasite and transmitted by the bite of an infective sand fly. Despite the importance of this disease there is no vaccine available for humans. Studies have shown that vector-transmitted infections are more virulent, promoting parasite establishment and abrogating protection observed against needle-injected parasites in vaccinated mice. KSAC and L110f, derived from *Leishmania*-based polyproteins, protected mice against the needle-injected parasites. Here, we tested the two molecules for their capacity to protect mice against cutaneous leishmaniasis transmitted by an infective sand fly. Our results show that KSAC, but not L110f, confers protection against *Leishmania* transmitted by sand fly bites where protection was correlated to a strong immune response to *Leishmania* antigens by memory T cells before and after sand fly transmission of the parasite. This is the first report of a *Leishmania*-based vaccine that confers protection against a virulent sand fly challenge. Our results support the importance of screening *Leishmania* vaccine candidates using infective sand flies before moving forward with the costly steps of vaccine development.

## Introduction

Leishmaniasis is a neglected disease endemic in 98 countries with an estimated 350 million people at risk and an estimated burden of 2,357,000 disability-adjusted life years [1]. Visceral leishmaniasis is fatal if left untreated, and the morbidity and stigma caused by cutaneous leishmaniasis is significant [2]. Current treatment is dependent on long-term therapy with toxic drugs, most requiring parenteral administration and hospital supervision.

A vaccine against leishmaniasis is feasible because infection with certain species, including *L. major*, or exposure to live *Leishmania* (leishmanization) leads to a long-term protection in humans [3], [4],[5],[6],[7]. Unfortunately, there is no commercial vaccine available for humans despite the presence of an extensive list of vaccine candidates shown to be protective in various animal models [8]. With the exception of two vaccine candidates, a synthetic glycovaccine [9] and autoclaved *L. major*+CPG [10], all *Leishmania* vaccines tested to date were challenged with needle inoculation of the *Leishmania* parasite. L110f and KSAC, two fusion polyproteins, in various combinations with appropriate adjuvants were shown to confer strong protection against cutaneous and visceral leishmaniasis in mice following conventional needle challenge [11], [12]. None of these vaccines were challenged by infected sand fly bites, the natural route of transmission. For protection against *L. major*, the L110f and KSAC-containing vaccines were tested separately in susceptible BALB/c mice followed by an infected sand fly challenge.

Both susceptible and resistant mice strains have been used to study the immunology of leishmaniasis and the protective effect of potential *Leishmania* vaccine candidates [13], [14], [15]. It has been long established that protection from *Leishmania* parasites requires the induction of a Th1 immune response [16], [17], [18]. BALB/c mice produce a polarized Th2 type immune response against *Leishmania* spp. and are used extensively to test *Leishmania* antigens [19]. It has been hypothesized that protective antigen/adjuvant formulations in this model system are good vaccine candidates since they have to overcome the natural Th2 bias of this strain.

Recently, Peters et al. [20] demonstrated that transmission of *Leishmania* parasites by sand fly bites generates a specific innate immune response involving a sustained recruitment of neutrophils that promotes parasite establishment. Additionally, the authors demonstrated that vector transmission of *Leishmania* parasites can abolish protection observed in vaccinated mice following needle challenge [10].

In the current work, we use a natural sand fly challenge model in BALB/c mice to test the immunogenicity and protective efficacy of the two fusion proteins L110f and KSAC formulated with GLA-SE against *L. major* transmitted by the bite of its natural sand fly vector *Phlebotomus duboscqi*.

## Materials and Methods

### Animals

We used 6 to 8 week old female BALB/c mice (Charles River Laboratories Inc). *Phlebotomus duboscqi* sand flies, Mali strain, were reared at the LMVR, NIAID, NIH.

### Ethics statement

All animal experimental procedures were reviewed and approved by the National Institute of Allergy and Infectious Diseases Animal Care and Use Committee under animal protocol LMVR4E. The NIAID DIR Animal Care and Use program complies with The Guide for the Care and Use of Laboratory Animals and with the NIH OACU ARAC guidelines.

### Parasites

We used the *L. major* V1 (MHOM/IL/80/Friedlin) strain for all sand fly infections apart from that shown in supporting information (Figure S1). In Figure S1, we used the WR 2885 strain, a recent field isolate that originated in Iraq and was typed at the Walter Reed Army Institute of Research. Washed amastigotes were counted and added to the blood meal for sand fly infection or placed directly in culture for the generation of metacyclics for needle challenge.

### Production of recombinant KSAC, L110f and GLA-SE

KSAC and L110f recombinant proteins were prepared as previously described [11], [12], [21] and mixed at the time of injection with a stable emulsion (SE) formulation of the pure, synthetic hexa-acylated TLR4 agonist glucopyranosyl lipid A (GLA) [22]. More than one lot of each antigen was used in experiments with a residual endotoxin content ranging from 77 to 245 EU/mg protein for L110f and from <0.05 to 27 EU/mg protein for KSAC.

### Vaccination of mice

Mice were vaccinated subcutaneously (s.c.) in the base of tail, three times at three week intervals with 100 µl containing 10 µg of antigen (KSAC or L110f) formulated with 20 µg of GLA-SE or with GLA-SE alone.

### Sand fly infection and transmission of *L. major* to vaccinated mice

Blood containing 3×10^6^ *L. major* amastigotes/ml was used to artificially feed sand flies as previously described [23]. Sand flies were used for transmission 13–14 days post-*Leishmania* infection. Three weeks (early challenge) or 12 weeks (delayed challenge) after the last immunization, 10 infected sand flies were applied to a single mouse ear for 2 hours in the dark.

### Intradermal needle challenge with *L. major* parasites

Vaccinated animals were injected intradermally in the right ear with 2×10^3^ purified *L. major* metacyclics in 10 µl PBS using a 27-gauge needle.

### Measurement of *Leishmania* cutaneous lesions

The thickness of ear lesions was recorded on a weekly basis using a vernier caliper (Mitutoyo Corp.).

### Parasite quantification by Real Time PCR

Parasite quantification was performed using JW11 and JW12 *Leishmania*-specific primers [24] as well as the 18S primers to amplify a housekeeping gene as previously described [25]. Expression levels were normalized to 18S DNA and corrected for the weight of the whole ear.

### Antibody detection by ELISA

Subclass (IgG1 and IgG2a) responses were measured by ELISA using Immulon4-Thermo plates coated overnight at 4°C with L110f or KSAC (2 µg/ml). Diluted sera (1/100) were incubated for 1 hour at 37°C. After washing, plates were incubated with alkaline phosphatase-conjugated anti-mouse IgG1 or IgG2a antibodies (BD Biosciences, San Jose, CA) (1/1000). The plates were developed using alkaline phosphatase substrate (SIGMA). The reaction was recorded after 10 minutes at 405 nm.

### Cytokine ELISA

Three weeks after the last immunization or one week post-challenge with infected sand fly bites, spleen cells were obtained as previously described [11] and stimulated with soluble *Leishmania major* antigen (SLA,100 µg/ml), KSAC (10 µg/ml) or L110f (10 µg/ml). Supernatants were collected 72 hours after incubation to evaluate cytokine production (IFN-γ, IL-10 and IL-4) by ELISA (BD Biosciences, San Diego, CA) according to the manufacturer's protocol.

### Flow cytometry

Three weeks after the last immunization or one week post-challenge with infected sand fly bites, 2×10^6^ splenocytes from individual mice were cultured in complete RPMI medium in flat-bottom 48-well plates with or without SLA (100 µg/mL), KSAC (20 µg/ml) or L110f (20 µg/ml) at 37°C in 5% CO_2_ for 18 h. Cells were incubated with Brefeldin A (BD Golgi Plug; BD Pharmingen) during the last 4 h of culture, washed with PBS, and blocked with anti-CD16/CD32 (BD Fc block, 2.4G2; BD Pharmingen) for 30 minutes at 4°C. Cells were stained with the fluorochrome-conjugated antibodies (BD Pharmingen and eBiosciences) PerCP-labeled anti-CD4 (RM4-5), APC-labeled anti-TCR-β (H57-597), PECy7-labeled anti-CD62L (MEL-14), and PE-labeled anti-CCR7 (4B12) for 30 minutes at 4°C, washed twice, fixed and permeabilized with Cytofix/Cytoperm Plus (BD Pharmingen), and stained with FITC-labeled anti-IFN-γ (XMG 1.2). A minimum of 100,000 cells were acquired using a FACS Calibur flow cytometer (BD Biosciences) and analyzed with the Flow Jo software (Tree Star, Inc., Oregon).

### Statistical analysis

A two-tailed unpaired Student's t-test was used for statistical analysis using the GraphPad software (GraphPad Software Inc.). P values of 0.05 or less were considered significant.

## Results

### Induction of antibodies in mice vaccinated with KSAC and L110f

**Figure 1:**
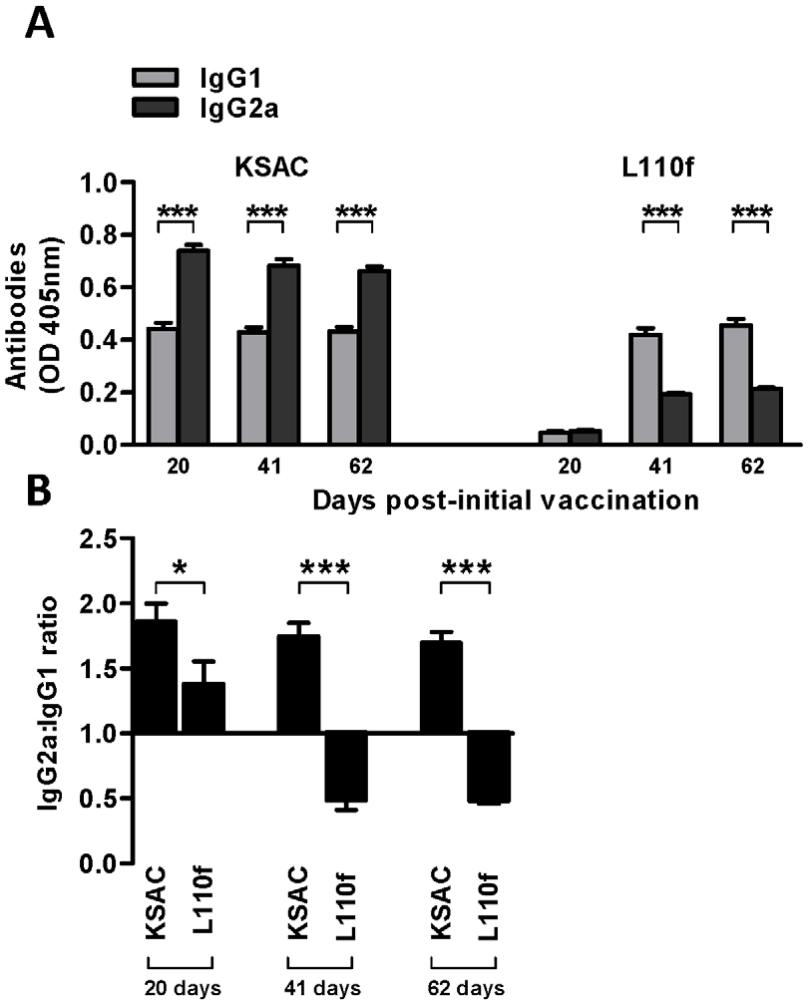
Antibody responses induced by vaccination with KSAC+GLA-SE or L110f+GLA-SE. BALB/c mice were vaccinated by the subcutaneous route three times every three weeks with either 20 µg adjuvant alone (GLA-SE), 10 µg KSAC+20 µg GLA-SE, or 10 µg L110f+20 µg GLA-SE. Serum was obtained one day before each immunization and 20 days after the last immunization. (A) KSAC- and L110f-specific IgG1 and IgG2a antibody levels in mice vaccinated with the respective antigen. (B) Ratio of antigen-specific IgG2a∶IgG1 levels in mice vaccinated with KSAC+GLA-SE or L110f+GLA-SE. The OD values are presented after subtraction of the OD value of mice vaccinated with GLA-SE alone. Ten mice were used in each group. Data are representative of two independent experiments.

Immunization of mice with KSAC+GLA-SE induced a robust antibody response following the first immunization and afterwards maintaining a positive IgG2a∶IgG1 ratio (Fig. 1 A, B). In contrast, L110f+GLA-SE induced a weaker overall antibody response that was biased towards IgG1 antibody production (Fig. 1A, B).

### Immunization with KSAC+GLA-SE protects against vector-transmitted *Leishmania major* (Friedlin V1) infection

**Figure 2:**
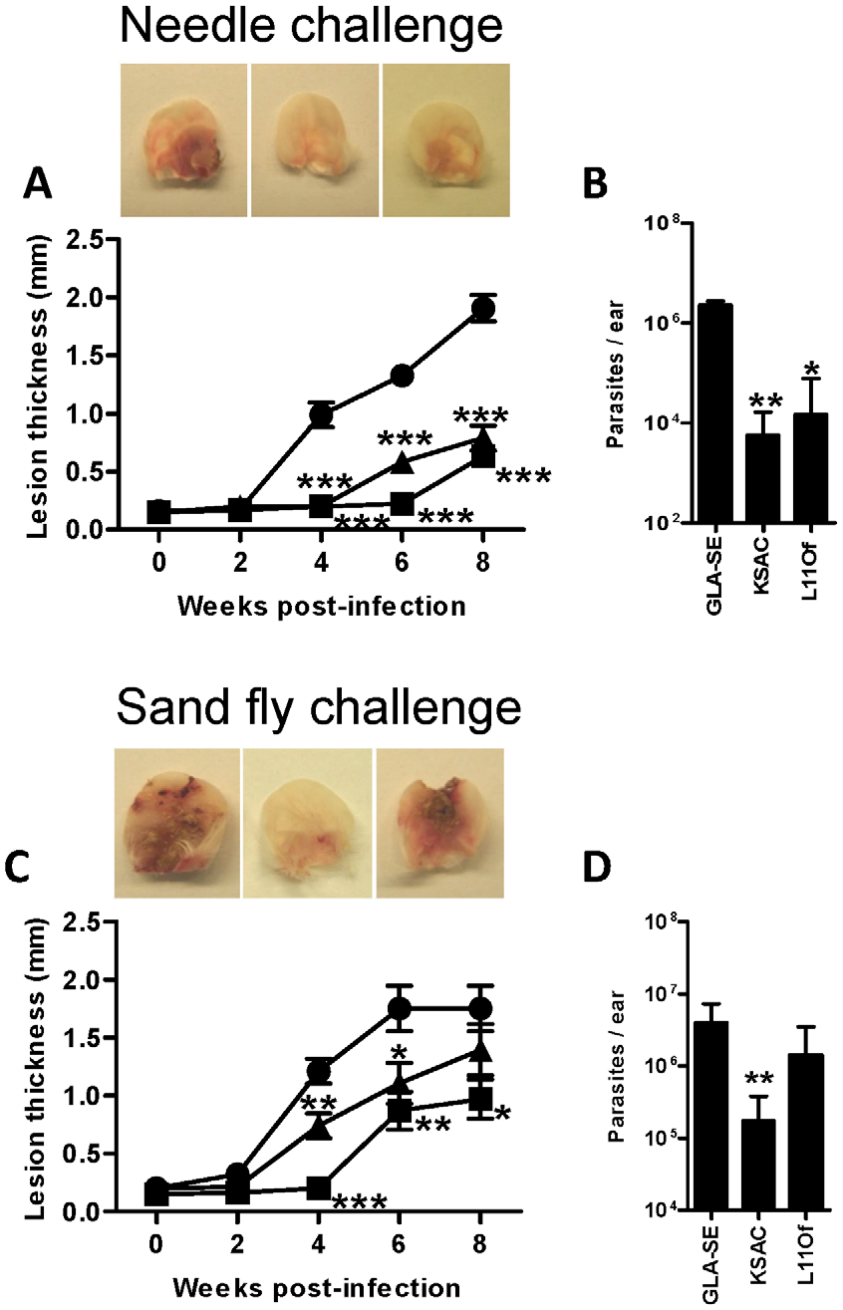
Vaccination of mice with KSAC+GLA-SE provides better protection from vector-transmitted *L. major* than does L110f+GLA-SE. BALB/c mice were challenged three weeks after the last vaccination in one ear with a needle injection of 2000 purified *L. major* metacyclics or exposed in one ear to bites of 10 *L. major*-infected sand flies (*P. duboscqi*). (A and C) Ear lesion thickness was measured in mice vaccinated with GLA-SE (•), KSAC+GLA-SE (▪), or L110f+GLA-SE (▴) and challenged with needle (A) or infected-sand fly bites (C). Panels show representative ears of vaccinated mice eight weeks after challenge. (B and D) Parasite load determined by Real-Time PCR eight weeks after challenge with needle (B) or infected-sand fly bites (D). Statistical significance was determined for KSAC+GLA-SE- or L110f+GLA-SE-vaccinated mice compared to the GLA-SE-vaccinated mice using a two-tailed, unpaired Student's t-test (*, p<0.05; **, p<0.01; ***, p<0.001). Five to seven mice were used in each group. Data are representative of three independent experiments.

Mice vaccinated with KSAC+GLA-SE or L110f+GLA-SE controlled a needle-challenge infection for at least eight weeks, whereas mice receiving GLA-SE alone did not (P<0.001, Figure 2A). Animals immunized with either vaccine also controlled *L. major* infection post-sand fly challenge through 6 weeks, although the level of protection with the L110f-containing vaccine was reproducibly lower than that for the KSAC-containing vaccine (Figure 2C). KSAC+GLA-SE, but not L110f+GLA-SLE immunized mice displayed significant protection up to the final 8 week time point (Figure 2C). The parasite burden assessed eight weeks after challenge with either needle or infected sand flies supports the pathology data (Figure 2B, D). Following needle challenge, the parasite number was significantly decreased in KSAC+GLA-SE (P<0.01) and L110f+GLA-SE (P<0.05) compared to GLA-SE immunized mice (Figure 2B). Following sand fly-challenge, only KSAC+GLA-SE immunized mice showed a significant reduction of parasite number (P<0.01) compared to GLA-SE immunized mice (Figure 2D). The number of parasites in L110f+GLA-SE immunized mice were intermediate, but showed no significant difference from controls (Figure 2D).

### Effector memory T cell responses in KSAC+GLA-SE and L110f+GLA-SE immunized mice pre- and post- challenge with *L. major* (Friedlin V1)-infected sand flies

**Figure 3:**
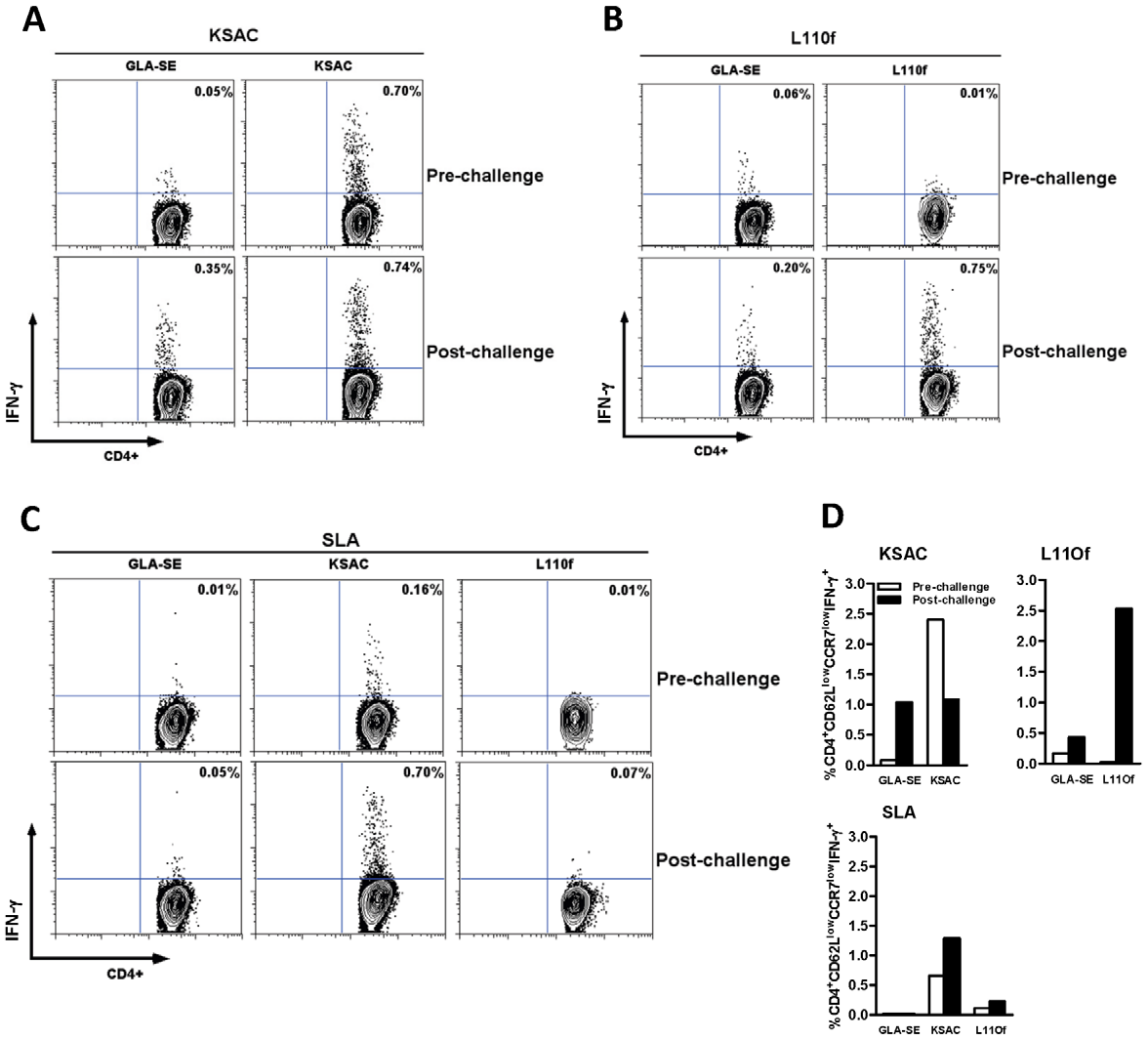
IFN-γ-producing CD4+ T cells before and after challenge with *L. major* -infected sand flies. Mice were vaccinated subcutaneously with 10 µg KSAC+20 µg GLA-SE, 10 µg L110f+20 µg GLA-SE, or 20 µg GLA-SE alone. Splenocytes were analyzed three weeks after the last immunization (Pre-challenge) or one week Post challenge with *L. major*. Splenocytes gated on TCRβ^+^CD4^+^ T cells for the percentage of IFN-γ^+^ CD4^+^ T cells produced after antigen stimulation (A–C). (A) Splenocytes from KSAC+GLA-SE- or GLA-SE-vaccinated mice stimulated with 20 µg recombinant KSAC *in vitro*; (B) Splenocytes from L110f+GLA-SE- or GLA-SE-vaccinated mice stimulated with 20 µg recombinant L110f; or (C) Splenocytes from KSAC+GLA-SE-, L110f+GLA-SE-, or GLA-SE-vaccinated mice stimulated with 100 µg SLA *in vitro*. (D) Splenocytes gated on TCRβ^+^CD4^+^CD62L^low^CCR7^low^ T cell population for the percentage of effector memory CD4^+^ splenic T cells in mice vaccinated with GLA-SE, KSAC+GLA-SE or L110f+GLA-SE after stimulation with either KSAC, L110f or SLA. Three mice were used per group. Data are representative of two independent experiments.

KSAC+GLA-SE vaccination induced the production of KSAC-specific IFN-γ^+^ CD4^+^ T cells, and the relative size of this cell fraction was maintained after a challenge with *L. major*-infected sand flies (Fig. 3A). In contrast, CD4^+^ T cells of mice immunized with L110f+GLA-SE and stimulated with L110f produced IFN-γ only after the mice were challenged with infected flies (Figure 3B). Importantly, CD4^+^ T cells from KSAC+GLA-SE-immunized mice produced IFN-γ following stimulation with SLA, while cells from animals immunized with L110f+GLA-SE were non-responsive (Figure 3C). Before sand fly challenge the percentage of CD4^+^CD62L^low^CCR7^low^ effector memory T cells producing IFN-γ was greater in KSAC-immunized mice stimulated *ex vivo* with KSAC compared to GLA-SE alone or L110f-immunized mice stimulated with L110f (Figure 3D, KSAC and L110f panels). Of note, the percentage of L110f-specific effector memory IFN-γ^+^CD4^+^ T cells from L110f+GLA-SE -immunized mice was greater after sand fly challenge than either the KSAC+GLA-SE or GLA-SE control groups stimulated with the appropriate antigens. Nevertheless, SLA induced a greater percentage of IFN-γ^+^ effector memory T cells from KSAC+GLA-SE-immunized mice than from either L110f+GLA-SE or the control GLA-SE groups before and after sand fly challenge (Fig. 3D, SLA panel).

### Antigen-specific IFN-γ predominates over IL-10 in vaccinated mice challenged with *L. major* (Friedlin V1)-infected sand flies

**Figure 4:**
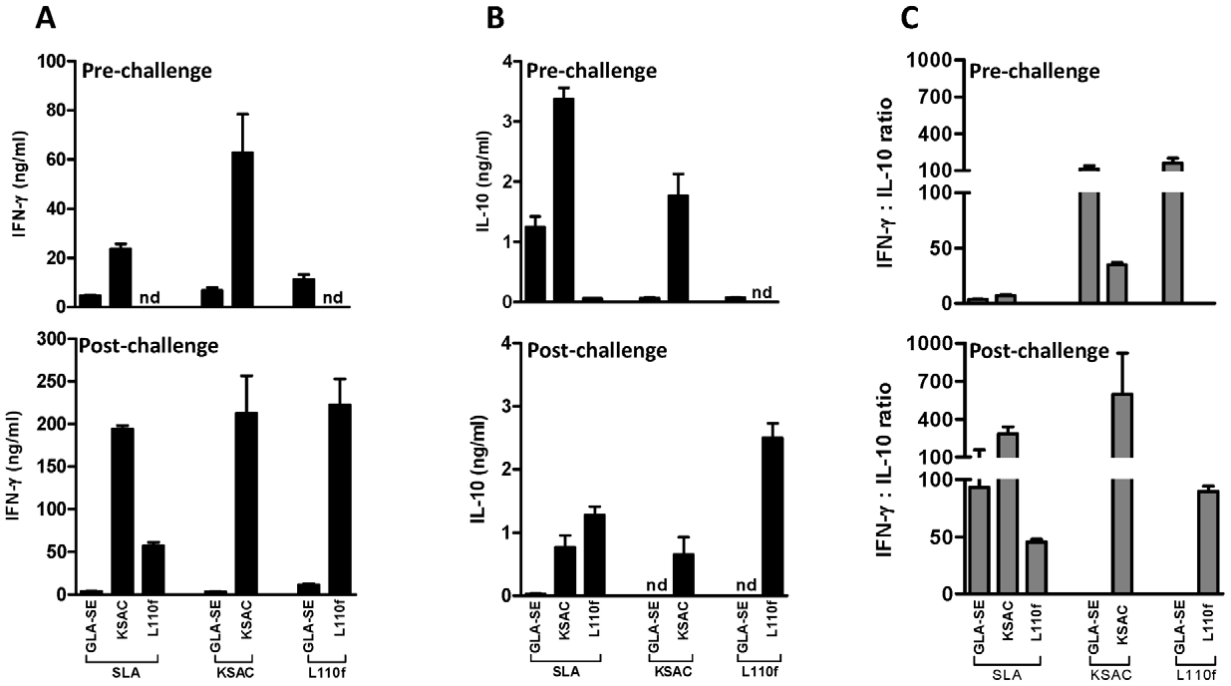
Cytokine production before and after challenge of mice with *L. major* -infected sand flies. Mice were vaccinated subcutaneously with 10 µg KSAC+20 µg GLA-SE, 10 µg L110f+20 µg GLA-SE, or 20 µg GLA-SE alone. IFN-γ (A) or IL-10 (B) production was measured by ELISA after *in vitro* stimulation of spleen cells with 100 µg SLA, 10 µg recombinant KSAC, or 10 µg recombinant L110f. Results are from stimulations performed at two time points: three weeks after the last immunization and one week post challenge. (C) Ratio of IFN-γ∶ IL-10 in vaccinated mice. Mean and SEM of three mice per group. Data are representative of two independent experiments. nd = none detected.

Mice vaccinated with KSAC+GLA-SE produced a high level of antigen-specific IFN-γ following ex vivo stimulation with SLA or KSAC both pre- and post-challenge with infected flies (Figure 4A). Prior to infected sand fly challenge, IFN-γ production was 23 ng/ml and 62 ng/ml following stimulation with SLA and KSAC, respectively (Figure 4A). This increased 8 and 3 fold when tested one week post-challenge, reaching 194 ng/ml and 212 ng/ml following stimulation with SLA and KSAC, respectively (Figure 4A). A distinctly different response was observed in L110f+GLA-SE-immunized mice where pre-challenge IFN-γ production was low to undetectable after stimulation with either SLA or L110f (Figure 4A), whereas IFN-γ was readily detectable after challenge with infected flies. Post-challenge, 57 ng/ml of IFN-γ and 222 ng/ml were produced in L110f-immunized mice in response to SLA and L110f, respectively (Figure 4A). Overall, antigen-stimulated splenocytes produced a low level of IL-10 compared to the relatively high concentration of IFN-γ produced (Figure 4A, B). The highest levels of IL-10 were produced by spleen cells of KSAC-immunized mice pre-challenge with infected flies (>3.3 ng/ml and 1.7 ng/ml with SLA and KSAC stimulation, respectively) compared to post-challenge levels (Figure 4A); post-challenge levels were reduced to <1 ng/ml following stimulation with either SLA or KSAC (Figure 4A). Similar to IFN-γ, IL-10 was not detectable in L110f+GLA-SE immunized mice pre-challenge and produced 1.2 and 2.4 ng/ml IL-10 after stimulation with SLA and L110f, respectively, after sand fly challenge (Figure 4A). The ratio of IFN-γ∶IL-10 highlight the dominance of IFN-γ over IL-10 in mice vaccinated with either KSAC+GLA-SE or L110f+GLA-SE and the lack of cytokine production in response to immunization with GLA-SE alone (Figure 4C).

### Immunization with KSAC+GLA-SE confers long-term protection against vector-transmitted *Leishmania major* (Friedlin V1) infection

**Figure 5:**
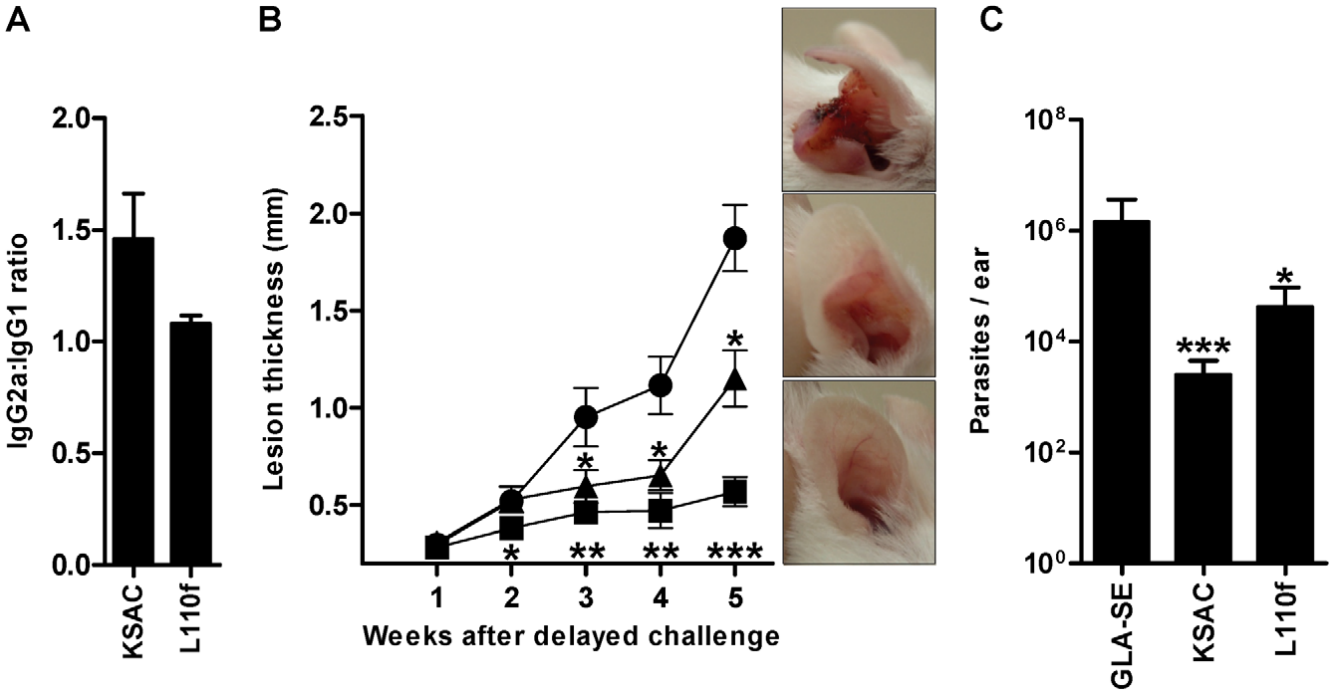
Vaccination with KSAC+GLA-SE confers long-term protection against *L. major* -infected *P. duboscqi* sand flies. Mice were vaccinated subcutaneously with 10 µg KSAC+20 µg GLA-SE, 10 µg L110f+20 µg GLA-SE, or 20 µg GLA-SE alone. (A) The ratio of IgG2a∶IgG1 was determined 12 weeks after the last vaccination and prior to the delayed challenge. (B) Lesion thickness in mice vaccinated with KSAC+GLA-SE (▪), L110f+GLA-SE (▴), or GLA-SE alone (•) and challenged with *L. major*-infected sand flies in the right ear 12 weeks (delayed challenge) after the last immunization. Panels show representative ears of vaccinated mice five weeks after challenge. (C) Parasite load determined by Real-Time PCR five weeks after challenge with infected-sand fly bites. Statistical significance was determined for mice vaccinated with KSAC+GLA-SE or L110f+GLA-SE compared to the adjuvant group using a two-tailed unpaired Student's t-test (*, p<0.05; **, p<0.01; ***, p<0.001). Ten mice were used per group. Data are representative of two independent experiments.

Mice vaccinated with KSAC+GLA-SE maintained a positive KSAC-specific IgG2a∶IgG1 ratio for at least 12 weeks after the final vaccination (Fig. 5A). These mice were protected against a delayed challenge with *L. major*-infected sand flies, maintaining significantly smaller lesions (P<0.05 to <0.001) compared to mice vaccinated with GLA-SE alone (Figure 5B). In this delayed challenge, mice vaccinated with L110f+GLA-SE exhibited an almost equal IgG2a∶IgG1 ratio lower than that of KSAC+GLA-SE vaccinated mice (Figure 5A), possibly reflecting a slower and less dramatic shift to a Th1 response. These mice were only partially protected against *L. major* transmitted by infected sand flies (Figure 5B). The long-term protection conferred by vaccination with KSAC is reflected by the absence of disease pathology up to five weeks post-challenge compared to GLA-SE-vaccinated mice and to a lesser degree to the partially protected L110f+GLA-SE-vaccinated mice (Figure 5B panels).

To assess the relevance of the parasite strain used in challenge, we carried out a preliminary experiment using a recent field isolate of *L. major* (WR-2885), a strain kindly provided to us by Dr. Edgar Rowton, Walter Reed Army Institute of Research, Washington, D.C., to test the level of protection provided by KSAC or L110f vaccines. KSAC-immunized mice were protected while L110f-immuinized mice lost the partial protection displayed against the Friedlin V1 laboratory attenuated parasite strain (Figure S1).

## Discussion

The *Leishmania*-derived polyproteins L110f and KSAC have been extensively studied. L110f or its first generation antigen Leish-111f (equivalent to the clinical antigen LEISH-F1) and KSAC elicited protective immunity against *L. major* as well as *L. infantum* in rodent models of infection initiated by needle challenge [11], [12], [26], [27]. Both antigens are well-defined and used in formulation with MPL®-SE or GLA-SE, adjuvants suitable for human use.

Here, we vaccinated BALB/c mice using the same protocol used by Bertholet et al. [11] for L110f and Goto et al. [12] for KSAC with the exception of the exclusive use of a stable emulsion containing GLA, a synthetic glucopyranosyl lipid A molecule similar to the naturally derived MPL® [21]. The objective was to test whether vaccination of mice with either antigen is equally protective against challenge by *L. major*-infected *P. duboscqi* sand flies. This work is relevant to the value of these antigens as vaccines; particularly following findings by Peters et al. [20] demonstrating that parasite transmission by vector bite induces a specific innate immune response, which promotes the establishment of *L. major* infection. The authors went further to demonstrate that the virulence of vector-transmitted infections overcomes the protection observed against a needle challenge of vaccinated mice [10].

When BALB/c mice were challenged with infected sand flies, the KSAC+GLA-SE-vaccinated animals were protected from infection by both a laboratory-maintained parasite and a more virulent strain that was recently isolated from a soldier in Iraq (WR-2885). The partial protection observed in L110f-vaccinated mice following either needle or fly challenge with the Friedlin-V1 strain was abrogated when the mice were challenged with sand flies carrying the virulent WR-2885 strain. These data are consistent with the reported increase in virulence of a vector-transmitted infection and emphasize the need to test promising vaccines in vector-transmission models [10], [20]. Additionally, the data draw attention to the importance of the virulence of the parasite strain used in challenge experiments particularly for vaccine studies.

It is important to point out that the clinical forms of L111f and L110f (LEISH-F1 and LEISH-F2, respectively) delivered with MPL®-SE were safe and immunogenic in healthy subjects with and without histories of previous infection with *L. donovani* [28]. Additionally, LEISH-F1 had some therapeutic value in patients with mucosal and cutaneous leishmaniasis where it appeared to shorten time to cure when used with chemotherapy [29], [30]. Both Leish-111f and Leish-110f demonstrated therapeutic efficacy in dogs with canine leishmaniasis [31], [32]. Therefore, L110f should not be overlooked as a valuable vaccine in our fight against leishmaniasis.

Based on present and previous data [12] KSAC and GLA-SE used together show considerable promise as a preventive vaccine. Vaccinated mice were mostly pathology-free after challenge with either the Friedlin V1 or WR2885 *L. major* strains. Additionally, vaccinated mice were protected in a delayed challenge 12 weeks after the last vaccination using infected sand flies, indicative of the generation of long-lasting immunity. Protection was associated with a consistently positive IgG2a∶IgG1 ratio for KSAC. Interestingly, in L110f-immunized mice that were partially protected, this ratio fluctuated from negative to neutral at 62 days and 12 weeks post-vaccination, respectively. Such antibody fluctuation may reflect stabilization of antibody levels over time and further emphasizes the importance of testing the efficacy of the immune response to a vaccine in a delayed challenge. Of note, both KSAC and L110f generated a Th1-biased cell-mediated immunity. This was demonstrated by the predominant antigen-specific IFN-γ response (relative to the IL-10 response) of spleen cells from vaccinated mice one week post-challenge. However, L110f-vaccinated mice did not mount an immune response to SLA nor to pre-challenge stimulation of spleen cells with antigen. This finding is distinct from that of Bertholet et al. [11] where they demonstrated a balanced IgG2a/IgG1 response to L110f plus GLA-SE or MPL-SE, a sizable induction of CD4^+^CD44^high^ IFN-γ^+^ cells, and good protection with both vaccines when challenged by needle. We cannot account with certainty for the apparent discrepancy between our immunogenicity results with L110f+GLA-SE and those of Bertholet et al. [11] and other reports using L111f/L110f+GLA-SE or +MPL-SE [26], [33], [34]. One difference between these studies and ours is the time chosen for pre-challenge analysis. In any case, the reduced protection we observed against the two *L. major* strains tested using L110f+GLA-SE correlate well with the relatively weak shift to a Th1 response that was observed after vaccination, but before parasite challenge. In contrast, splenocytes from KSAC+GLA-SE-vaccinated mice responded well to stimulation with antigen and, more importantly, to stimulation with SLA pre- and post- challenge by infected bites. KSAC-immunized mice generate a pool of effector memory CD4^+^IFN-γ^+^ T cells specific to KSAC that was efficiently stimulated with SLA both before and after a sand fly challenge. These results suggest that the rate and magnitude of the immune response are important for the generation of protection against a virulent sand fly-transmitted infection. Additionally, the observed differences in the protective effect of L110f and KSAC, both formulated with GLA-SE, against vector-challenge may be related to other factors such as antigenicity, accessibility or amount of the natural proteins making up these polypeptides.

Recent studies (S. Bertholet, personal communication) have shown that lower doses of adjuvant (GLA-SE) are more efficient at inducing long-lived CD4 memory responses, especially with L110f as an antigen (data not shown). This could indicate that optimal adjuvant doses vary for different antigens and might need to be titrated accordingly. The KSAC results reported here demonstrate that the synthetic TLR4 agonist GLA can be a powerful tool to direct a shift from Th2 to a Th1-type response, necessary to combat a vector-transmitted *L. major* infection.

In summary, the BALB/c mouse model was used in our experiments because it is especially susceptible to *L. major* infection as a result of its genetically determined Th2 immune response. We observed that immunization with KSAC in combination with the TLR4 agonist GLA in stable emulsion overcomes the Th2 bias of BALB/c mice, generating a robust, cell-mediated Th1 immune response in these mice. This immunological activation results in solid protection against vector-transmitted *L. major* infection, protection that is comparable to the one observed following needle challenge. As anticipated, immunization conditions (using L110f+GLA-SE) that produced a more modest immune response with a less dramatic shift from a Th2 to a Th1 response was less protective.

KSAC, a defined *Leishmania*-based vaccine candidate shows protection against a sand fly challenge, and this protection was produced in combination with the clinically viable adjuvant GLA-SE. With these encouraging results, more work is needed to test the protective nature of these vaccine components in more relevant models of cutaneous leishmaniasis.

## Supporting Information

Figure S1 **Protection in KSAC+GLA-SE-vaccinated mice following sand fly challenge with a recent** ***L. major*** **isolate.** Mice were vaccinated subcutaneously with 10 µg KSAC+20 µg GLA-SE, 10 µg L110f+20 µg GLA-SE or 20 µg GLA-SE alone and challenged 12 weeks later (delayed challenge) with sand flies infected with a highly virulent strain of *L. major* recently isolated from a human lesion. (A) Lesion thickness in mice vaccinated with KSAC+GLA-SE (▪), L110f+GLA-SE (▴), or GLA-SE alone (•). (#) Mice were euthanized 4 weeks post-challenge due to severity of the lesions. (B) Panels showing representative lesions on ears of mice vaccinated with GLA-SE (1), KSAC+GLA-SE (2), or L110f+GLA-SE (3) three, four, and five weeks post-delayed challenge with *L. major*-infected sand fly bites.(**Ψ**) Five weeks post-challenge, only one out of 5 mice showed a small ulcerated lesion in the group vaccinated with KSAC+GLA-SE. Statistical significance was determined for KSAC+GLA-SE- or L110f+GLA-SE-vaccinated mice compared to the adjuvant group using a two-tailed unpaired Student's t-test (*, p<0.05). Five mice were used per group. The experiment was carried out once.
(TIFF)
