# Supplementary figures and images for: KSAC, a Defined Leishmania Antigen, plus Adjuvant Protects against the Virulence of L. major Transmitted by Its Natural Vector Phlebotomus duboscqi
Source: PLoS Negl Trop Dis. 2012 Apr 3;6(4):e1610. doi: 10.1371/journal.pntd.0001610 (PMC3317914; doi:10.1371/journal.pntd.0001610)

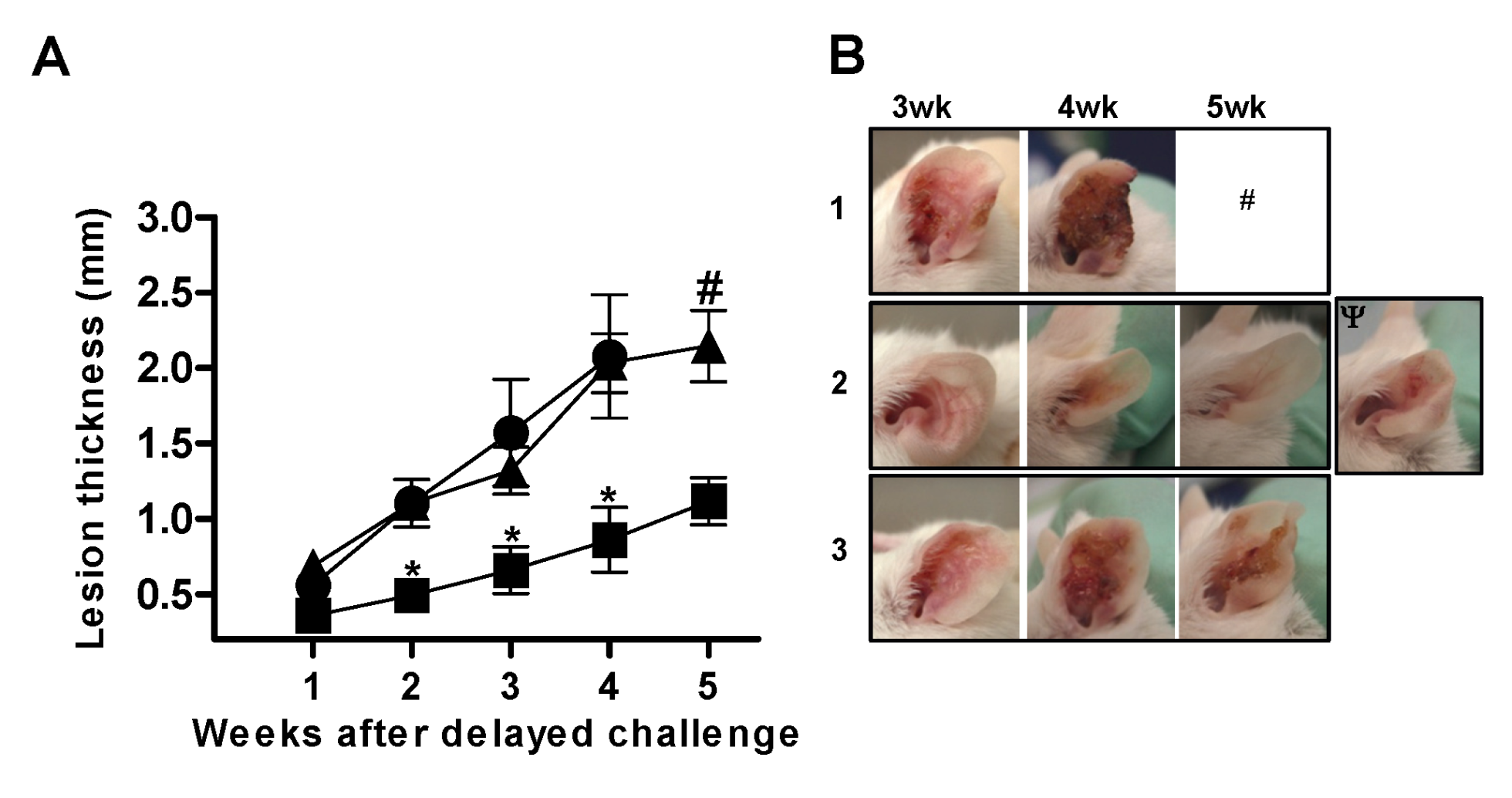

Supplement: Figure S1 — Protection in KSAC+GLA-SE-vaccinated mice following sand fly challenge with a recent L. major isolate. Mice were vaccinated subcutaneously with 10 µg KSAC+20 µg GLA-SE, 10 µg L110f+20 µg GLA-SE or 20 µg GLA-SE alone and challenged 12 weeks later (delayed challenge) with sand flies infected with a highly virulent strain of L. major recently isolated from a human lesion. (A) Lesion thickness in mice vaccinated with KSAC+GLA-SE (▪), L110f+GLA-SE (▴), or GLA-SE alone (•). (#) Mice were euthanized 4 weeks post-challenge due to severity of the lesions. (B) Panels showing representative lesions on ears of mice vaccinated with GLA-SE (1), KSAC+GLA-SE (2), or L110f+GLA-SE (3) three, four, and five weeks post-delayed challenge with L. major-infected sand fly bites.(Ψ) Five weeks post-challenge, only one out of 5 mice showed a small ulcerated lesion in the group vaccinated with KSAC+GLA-SE. Statistical significance was determined for KSAC+GLA-SE- or L110f+GLA-SE-vaccinated mice compared to the adjuvant group using a two-tailed unpaired Student's t-test (*, p<0.05). Five mice were used per group. The experiment was carried out once. (TIFF) [file pntd.0001610.s001.tif]
